# Proteomics Analysis Reveals Diverse Molecular Characteristics between Endocardial and Aortic-Valvular Endothelium

**DOI:** 10.3390/genes12071005

**Published:** 2021-06-30

**Authors:** A. Aneesh Kumar, G. S. Ajith Kumar, Gopika Satheesh, Arun Surendran, Mahesh Chandran, Chandrasekharan C. Kartha, Abdul Jaleel

**Affiliations:** 1 Cardiovascular Diseases and Diabetes Biology, Rajiv Gandhi Centre for Biotechnology, Thiruvananthapuram 695014, India; aneeshkumar@rgcb.res.in (A.A.K.); ajithnair@rgcb.res.in (G.S.A.K.); gopikasatheesh@rgcb.res.in (G.S.); cckartha@rgcb.res.in (C.C.K.); 2 Mass Spectrometry and Proteomics Core Facility, Rajiv Gandhi Centre for Biotechnology, Thiruvananthapuram 695014, India; arunsurendran@rgcb.res.in (A.S.); maheshchandran@rgcb.res.in (M.C.)

**Keywords:** aortic valvular endothelium, endocardial endothelium, label-free proteomics, pathway analysis

## Abstract

The variations in the protein profile of aortic-valvular (AVE) and endocardial endothelial (EE) cells are currently unknown. The current study’s objective is to identify differentially expressed proteins and associated pathways in both the endothelial cells. We used endothelial cells isolated from the porcine (*Sus scrofa*) aortic valve and endocardium for the profiling of proteins. Label-free proteomics was performed by liquid chromatography-tandem mass spectrometry (LC-MS/MS). Our proteomics analysis revealed that 29 proteins were highly expressed, and 25 proteins were less expressed in the valve than the endocardial endothelium. The cell surface markers, such as CD63, ICAM1, PECAM1, PROCR, and TFRC, were highly expressed in EE. In contrast, CD44 was highly expressed in AVE. The pathway analysis showed that metabolic process-related proteins and extracellular matrix-related proteins were enriched in valves. Differential enrichment of signaling pathways was observed in the endocardium. The hemostasis function-related proteins were increased in both endothelial cells. The proteins and pathways enriched in aortic-valvular and endocardial endothelial cells revealed the distinct phenotype of these two closely related cells.

## 1. Introduction

A monolayer of endothelial cells covers the entire cardiovascular system, including aortic valves and cardiac chambers. The differential expression analysis of proteins can reveal the phenotypic uniqueness and similarities of endothelial cells isolated from anatomically different locations. Endothelial cells have a role in regulating functions such as coagulation and fibrinolysis, inflammatory responses, lipoprotein metabolism, vascular tone, and hormone metabolism [1]. The endothelium has a vital role in the pathogenesis of valve-associated heart diseases [2]. The abnormal morphology of valvular endothelial cells affects the thrombogenicity, the synthetic function, and intracellular permeability of the valves. For instance, a bacterial infection of valves disrupts endothelial barriers and leads to infective endocarditis. *Staphylococcus aureus* is the primary causative agent for infective endocarditis [3]. An infection with β-hemolytic streptococci induces the release of autoantibodies that react with human tissue antigens such as collagen, which leads to rheumatic fever. The pathology of rheumatic fever is associated with scars observed only in the endothelium of cardiac valves and not in other affected tissues [4]. The thickening and calcification of aortic valves are defined as aortic valve sclerosis (AVS). AVS is also associated with endothelial dysfunction [5].

The previous studies showed that valvular endothelium has a distinct phenotype compared to arterial endothelial cells by transcriptome analysis [6]. A differential transcriptome profile of valvular endothelium isolated from both sides of the porcine aortic valve leaflets was reported by Simmons et al. [7]. The unique phenotype of valvular endothelial cells compared to vascular endothelial cells can be related to the fact that valvular endothelium is derived from the endocardial endothelial cells during embryonic development, unlike vasculogenesis [8].

The inner cavity heart chamber of the heart is covered by endocardial endothelial cells (EE) [9]. Previous studies reported that EE cells are derived from a specific group of progenitor cells that are distinct from hemangioblasts, contributing to the formation of blood vessels [10]. In the present, we compared the expression of proteins in endothelial cells isolated from valves and endocardium. We had previously reported several differentially expressed proteins and pathways in the endocardial endothelium compared to the aortic endothelium [11]. For instance, a protein involved in preventing thrombus formation called endothelial protein C receptor (PROCR) was overexpressed in the endocardium. Pathway analysis showed that stress-response-related proteins were enriched in the endocardium compared to the aorta. However, the proteome level characterization of valvular endothelial cells compared to endocardial endothelial cells was not reported previously.

The current study’s objective is to characterize the differences in the protein profile of endothelial cells isolated from the aortic valve and endocardium of the porcine heart. The proteome profile showed that the protein expression in the aortic valvular endothelium is unique when compared to the endocardial endothelium.

## 2. Materials and Methods

### 2.1. Endothelial Cell Culture and Protein Isolation

The pig (*S. scrofa*) heart samples (*n* = 3) were collected from a local slaughterhouse for the current study. The ethical approval was obtained from the institutional animal ethics committee of Rajiv Gandhi Centre for Biotechnology. The excised heart samples were harvested in phosphate-buffered saline (PBS). An antibiotic-antimycotic cocktail (15240, Gibco-Life Technologies, New York, NY, USA) and 100 U/mL heparin (H3149, Sigma-Aldrich, St. Louis, MO, USA) also added to the PBS. An enzymatic method with 0.2% collagenase type-2 (C6885, Sigma-Aldrich) was used to isolate endothelial cells from the endocardium and aortic valve [11,12]. The isolated cells were cultured in a 60 × 15 mm culture dish (353002, Falcon, Scottsdale, AZ, USA). The MCDB 131 with 20% fetal bovine serum was used for the cell culture at 37 °C and 5% CO_2_. The cells were harvested after reaching the confluence, and pellets were stored at −80 °C.

The RapiGest^TM^ SF surfactant (Waters, Manchester, UK) in 50 mM ammonium bicarbonate was used to isolate proteins from the cell pellets. The bicinchoninic acid assay (BCA) was used for the estimation of the protein concentration. In-solution trypsin digestion was performed in 100 µg (100 µL, concentration—1 µg/µL) proteins. The DL-Dithiothreitol (DTT, 5 µL, stock concentration—100 mM) was used for the reduction of disulfide bonds in proteins. The alkylation was conducted with (5 µL, stock concentration—200 mM) iodoacetamide (IAA) at room temperature in the dark for 30 min. The proteins were digested with trypsin (Sigma) by incubating overnight at 37 °C. The 100% formic acid (1 µL) was used for the arresting of trypsin digestion. The whole reaction mixture has 100 µg/100 µL protein + 5 µL of DTT + 5 µL of IAA + 10 µL of trypsin (0.04 µg/µL) + 1 µL of formic acid. Thus, the final concentration of peptides would be 0.83 µg/µL in all samples. The digested peptides were centrifuged at 14,000 rpm for 12 min, and the supernatants were used for the LC/MS/MS analysis.

### 2.2. Liquid Chromatography and Mass Spectrometry

A nano ACQUITY UPLC System (Waters) chromatography system coupled to a Quadrupole Time-of-Flight (Q-TOF) mass spectrometer (SYNAPT-G2, Waters) were used for the LC/MS/MS analysis. Each sample was analyzed three times (three technical replicates). The Mass Lynx 4.1 software was used for the control and operation of both instruments. Three microliters of peptides were injected for the measurement. A trap column (Symmetry^®^ 180 μm × 20 mm C18 5 μm, Waters) was used to remove the salts from the sample. A 75 μm × 100 mm BEH C18 Column (Waters), with a particle size of 1.7 μm was employed for the peptide separation. The chromatography column was maintained at 40 °C. The mobile phase-A was water, and the mobile phase-B was acetonitrile; both contain 0.1% formic acid. Gradient elution of mobile phase-B (1–40%) was employed for 55.5 min. The column was washed with 80% mobile phase-B for 7.5 min. One percent mobile phase B was used for the re-equilibration of the column. The flow rate of 300 nL/min was used throughout the run.

The data were acquired in continuum format in elevated collision energy (MS^E^) mode with ion mobility. The resolving power of 18,000 FWHM in resolution mode was used for the acquisition of data. Data were collected for 0.9 s with an interscan delay of 0.024 s. [Glu^1^]-Fibrinopeptide B human (Sigma) was used for the lock mass acquisition every 30 s at a flow rate of 500 nL/minutes. The proteomics data were uploaded in the ProteomeXchange Consortium via the PRIDE partner repository with the dataset identifier PXD025922. The previously published endocardial endothelium proteomics data set was used to compare the aortic valvular endothelial cells in this study [11].

### 2.3. Data Analysis of Proteomics Data

Progenesis QI for Proteomics (Non-linear Dynamics, Newcastle upon Tyne, UK) was used for the analysis of proteomics data. The *S. scrofa* database downloaded from the NCBI was used for the annotation of peptides. We have used the default protocol provided in the Progenesis website for the data analysis (https://www.nonlinear.com/progenesis/qi-for-proteomics/, accessed on 28 April 2021). Briefly, an automatic sensitivity method based on a noise estimation algorithm was used for the determination of noise in the data for the peak picking of the data. A maximum ion charge of 20 was used. A false discovery rate of 4% was used for the search. The detection of at least one unique peptide was used for the identification. The carbamidomethylation of cysteine and oxidation of methionine were selected as fixed and variable modifications, respectively. A specificity of one missed cleavage was used for the trypsin digestion parameter during database search. Normalized intensity values obtained from three biological replicates samples were used for the label-free quantification analysis. The Student’s *t*-test was used for the differential expression analysis using Progenesis QI for Proteomics. A protein with a raw *p*-value < 0.05 and fold change > 1.5 was considered significant. An adjusted *p*-value (False Discovery Rate or *q*-value) < 0.25 was used to select significantly changed proteins since the number of differentially expressed proteins was markedly less in this study.

A WEB-based GEne SeT AnaLysis Toolkit (WebGestalt) was used to identify significantly (*p*-value < 0.05) enriched pathways associated with altered proteins [13]. The gene set enrichment analysis (GSEA) was used to identify the cell differentiation markers [14]. A web-based tool called Heatmapper was used to construct Heatmap [15]. The GraphPad Prism software was used for plotting cell differentiation markers. The FunRich tool was used for the construction of the Venn diagram [16]. Finally, the volcano plot was prepared using VolcaNoseR software [17].

## 3. Results

**Figure 1:**
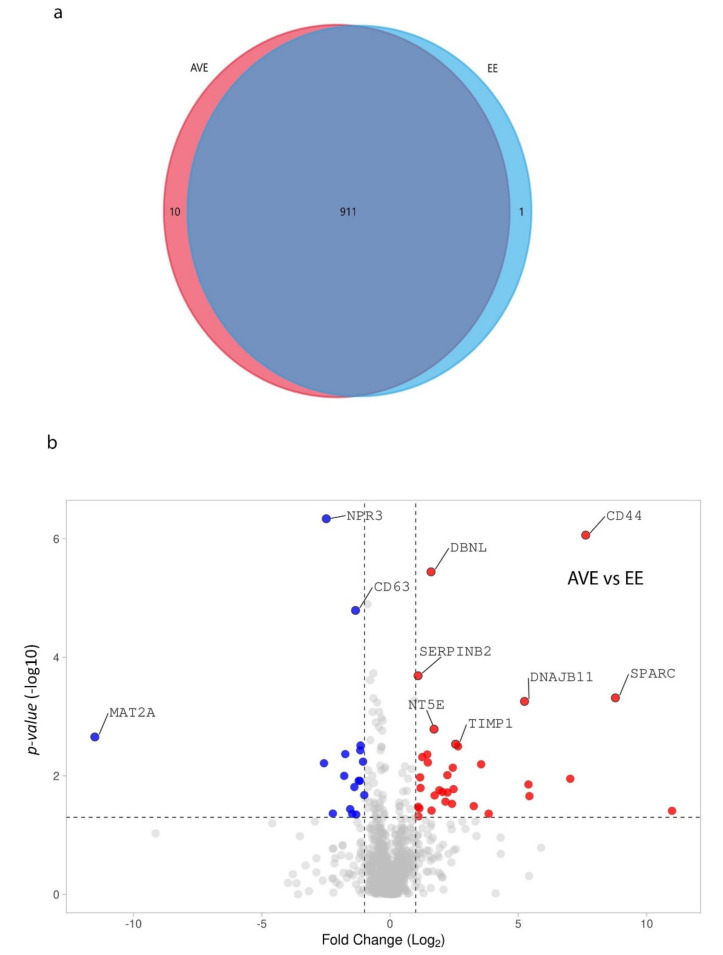
Venn diagram represents (**a**) the distribution of all detected proteins in aortic valvular endothelium (AVE) and endocardial endothelium (EE). The volcano plot represents (**b**) differentially expressed proteins in AVE cells compared to EE cells. The Student’s *t*-test was used for the differential expression analysis using Progenesis QI for Proteomics. Proteins with a raw *p*-value < 0.05 and fold change > 2 were highlighted in the figure (Red—upregulation and Blue—down-regulation).

**Figure 2:**
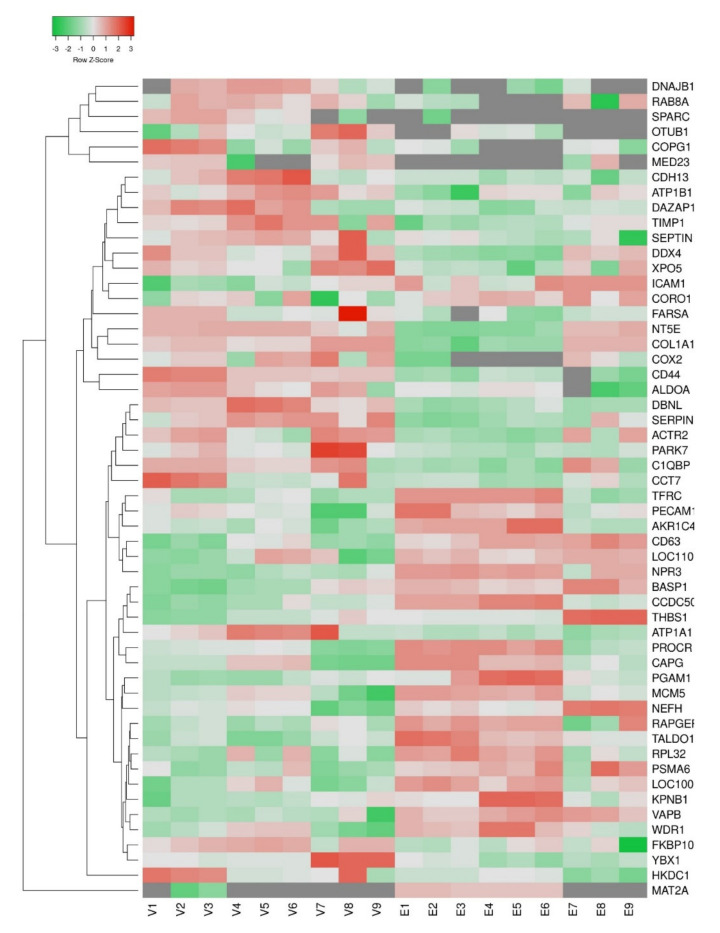
Heatmap represents differentially changed proteins in aortic valvular and endocardial endothelium. The normalized peptide intensities from label-free proteomics data were used to create the Heatmap using the Heatmapper tool. Supplementary Table S1. Fold change > 1.5 was considered significant. V—Aortic Valvular endothelial cells; E—Endocardial endothelial cells.

We identified 922 proteins in aortic-valvular (AVE) and endocardial endothelial (EE) cells by proteomics analysis. The overall distribution showed that 911 proteins were commonly observed in both types of endothelial cells (Figure 1a). The differential expression analysis showed that 29 proteins were significantly overexpressed in AVE cells compared to EE cells. Similarly, 25 proteins were overexpressed in EE cells compared to AVE cells (Figure 1b, and Figure 2; Supplementary Table S1).

**Figure 3:**
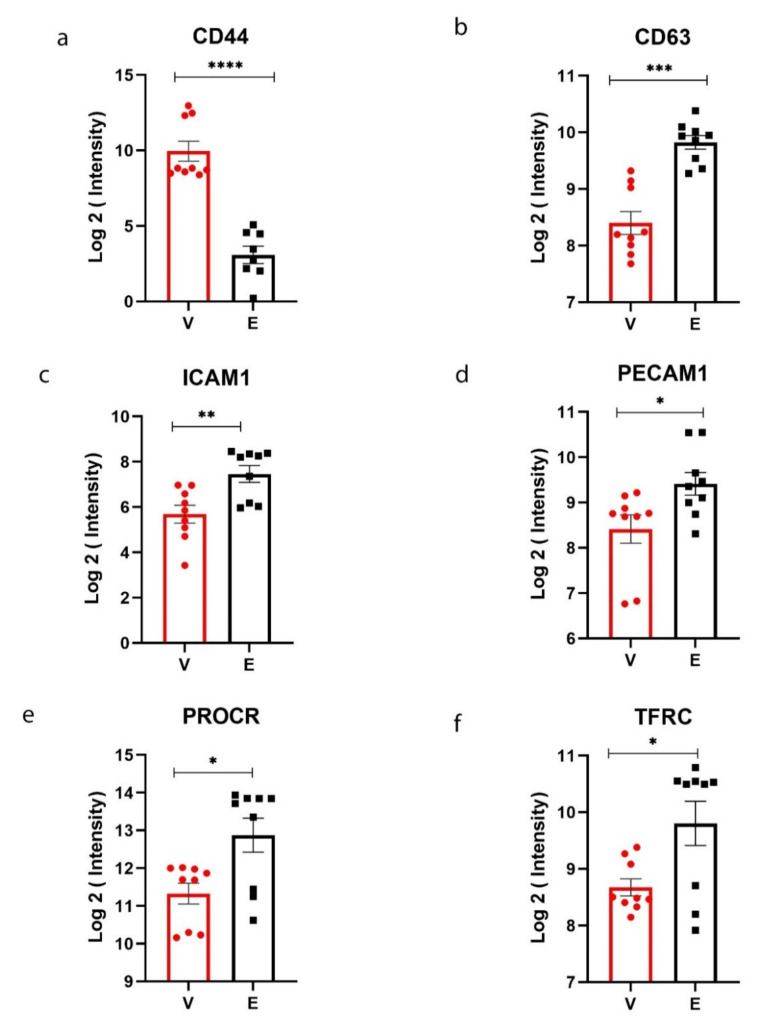
Cell surface markers identified in aortic valvular (AVE)and endocardial endothelium (EE). The GSEA analysis was used to determine the cell differentiation (CD) markers. The CD-marker highly expressed in AVE cells was represented in (**a**). The CD-markers highly expressed in EE cells were shown in (**b**–**f**). Cell The normalized peptide intensities from label-free proteomics data were used to create the graphs. The Student’s *t*-test was used for the differential expression analysis using Progenesis QI for Proteomics. A protein with a raw *p*-value < 0.05 and fold change > 1.5 was considered significant. V—Valvular endothelial cells; E—Endocardial endothelial cells. * *p*-value < 0.05, ** *p*-value < 0.01, *** *p*-value < 0.001 and **** *p*-value < 0.0001.

The GSEA analysis showed that cell differentiation marker protein called CD44-antigen (CD44) is significantly overexpressed in AVE cells compared to the EE cells. However, marker proteins such as CD63-antigen (CD63), intercellular adhesion molecule-1 (ICAM1), platelet endothelial cell adhesion molecule (PECAM1), endothelial protein C receptor (PROCR), and transferrin receptor protein-1 (TFRC) were highly expressed in endocardial endothelial cells (Figure 3 and Supplementary Figure S1).

**Figure 4:**
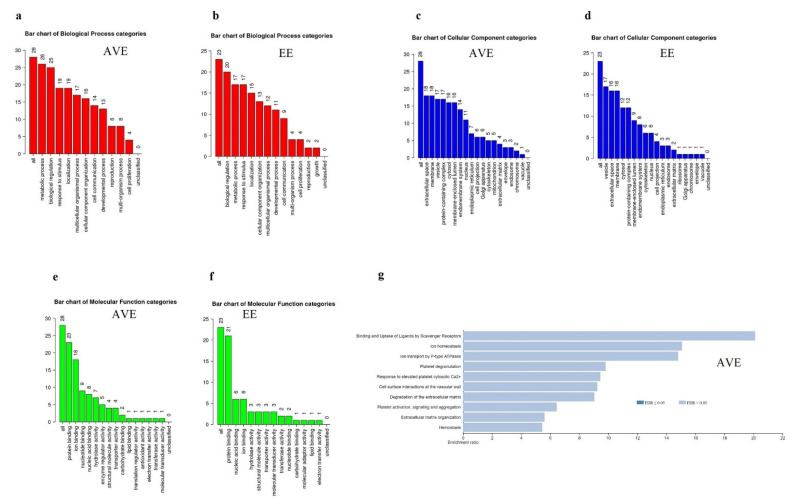

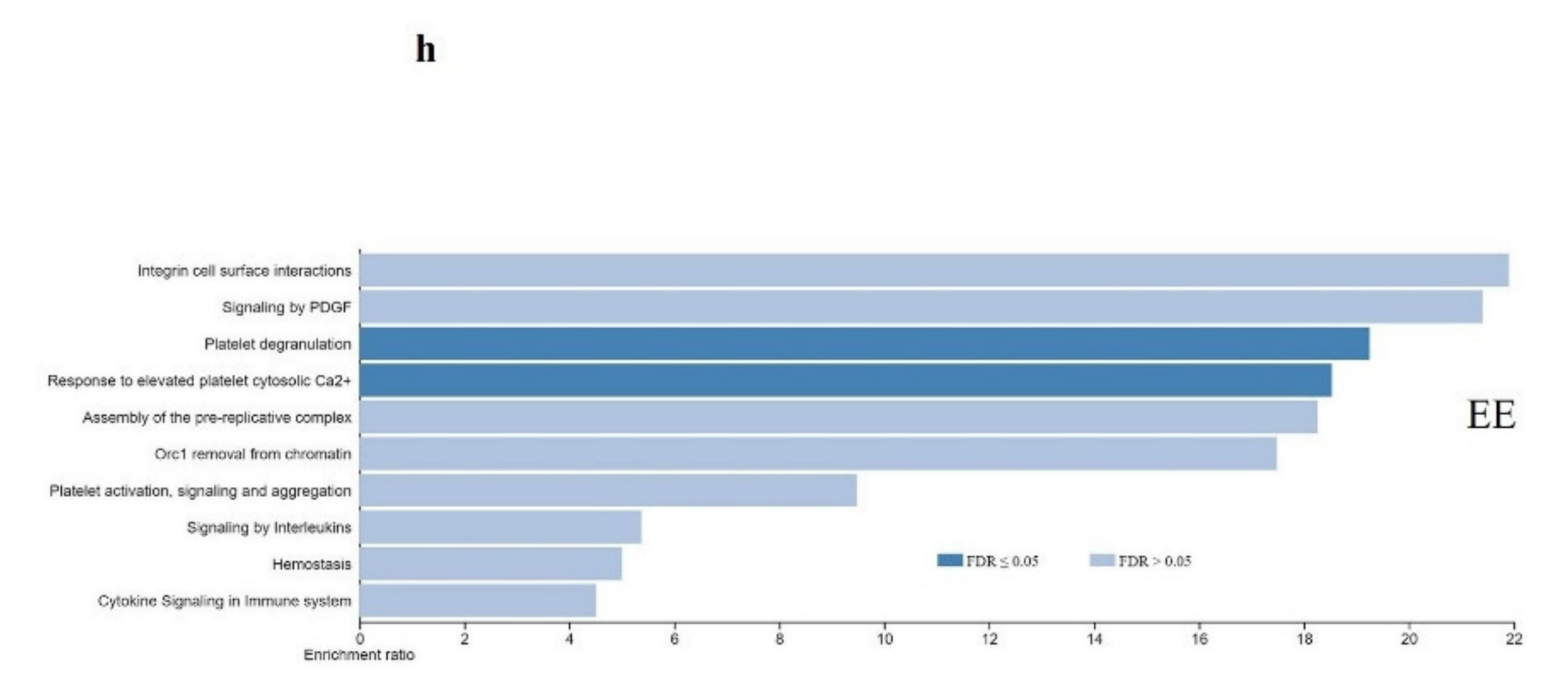
Classification of proteins overexpressed in aortic valvular endothelium (AVE) and endocardial endothelium (EE). A list of proteins in AVE cells (29 genes) was used for the analysis, 28 genes were mapped to unique Entrez gene IDs, and 1 gene symbol was not mapped to any IDs. A list of proteins in EE cells (25 genes) was used for the analysis, 23 genes were mapped to unique Entrez gene IDs, and 2 gene symbols were not mapped to any IDs. The bar chart represents Entrez gene IDs overlapped with gene ontology (GO) slim categories such as biological process (**a**,**b**), cellular component (**c**,**d**), and molecular function (**e**,**f**). The bar chart represents the top 10 enriched pathways in AVE cells and EE cells using the Reactome database (**g**,**h**). The darker bar represents FDR for less than or equal to 0.05, while the lighter shade represents FDR > 0.05. The top 10 pathways with a *p*-value < 0.05 are selected for the representation. All classification analysis was performed in WebGestalt software.

The WebGestalt analysis was performed to identify significantly enriched pathways in AVE and EE cells (Figure 4) using differentially expressed proteins in both groups. The gene ontology analysis showed that proteins related to the metabolic process are upregulated in AVE cells (Figure 4a). In contrast, proteins related to biological regulations are upregulated in EE cells (Figure 4b). The classification of proteins based on cellular component categories showed that many of the proteins overexpressed in AVE cells (Figure 4c) are associated with the extracellular matrix. Vesicle-related proteins were overexpressed in EE cells (Figure 4d) compared to AVE cells. Molecular function categories showed that different types of protein-binding proteins were enriched in both endothelial cells. A significantly greater number of ion binding proteins were overexpressed in AVE cells than EE cells (Figure 3c and Figure 4c). A pathway analysis using the Reactome database showed that proteins related to hemostasis, platelet activation-signaling and aggregation, platelet degranulation, and response to elevated platelet cytosolic Ca^2+^ were enriched in both endothelial cells (Figure 4e,f). Proteins related to pathways such as cell surface interactions at the vascular wall, degradation of the extracellular matrix, binding and uptake of Ligands by scavenger receptors, extracellular matrix organization, ion homeostasis, and ion transport by P-type ATPases were overexpressed in AVE cells compared to EE cells (Figure 4g). However, the overexpressed proteins identified in EE cells are related to unique pathways such as integrin cell surface interactions, cytokine signaling in the immune system, signaling by PDGF, assembly of the pre-replicative complex, signaling by interleukins, and Orc1 removal from chromatin (Figure 4h).

## 4. Discussion

The label-free proteomics analysis of endothelial cells isolated from the aortic valve showed a significantly different phenotype than the endocardial endothelial cells. A restricted portion of endocardial endothelial cells acts as the progenitor of the valve cells during the embryonic development of the vertebrate heart [18]. The functional role and morphological differences of both endothelial cells can be related to the differential proteome identified in the current study. However, both kinds of cells have a closely associated developmental origin.

The proteome analysis showed that only 54 proteins out of 922 detected proteins were differentially expressed in aortic valvular endothelial cells than endocardial endothelial cells. This smaller number of differential expression of proteins can be associated with their common developmental origin and proximity. Cell differentiation markers are essential for the identification and characterization of specific cell types. We have identified that the CD-44-antigen is significantly overexpressed in AVE cells. The CD-44 antigen is involved in the adhesion of lymphocytes to human endothelial cells [19]. The CD-44 protein could be an ideal marker for future characterization studies specific to AVE cells. A previous study reported that elevated levels of CD-44 are associated with fibroblastic phenotype in cultured human corneal endothelial cells [20]. CD-44 is a cell-surface receptor for hyaluronan, involved in cell-matrix and cell–cell adhesions [21].

Cell surface markers, such as CD-63 antigen, ICAM1, PECAM1, PROCR, and TFRC, were overexpressed in endocardial endothelial cells. The CD-63 antigen is involved in platelet activation [22]. Previous studies also showed that endothelial-leukocytes adhesion molecules such as ICAM1 and PECAM-1 are highly expressed in the endocardium [23]. The platelet endothelial cell adhesion molecule (PECAM)-1 is also an excellent marker for tracking vascular formation and early heart development [24]. The endothelial protein C receptor (PROCR) plays a crucial role in modulating antithrombotic pathways since endothelium was considered a thrombo-resistant surface [25]. A reduced expression of PROCR was reported in the heart failure mice model with endocardial endothelial dysfunction [26]. The TFRC protein is involved in the cellular uptake of iron by endocytosis essential for iron homeostasis. Previous studies reported that transferrin receptors are present in blood capillaries in the brain that allow transferrin and iron into brain tissues [27].

The gene ontology analysis showed that proteins involved in the metabolic process are enriched in AVE cells. The differential expression of proteins involved in the metabolic process in AVE cells can be linked to the constant movement of the valve and increased stress and strain on the valvular endothelial cells compared to the endocardium [28]. We observed the increased expression of extracellular matrix (ECM) related proteins in AVE cells. A previous RNA expression study also showed the enrichment of ECM-related genes in the valve of domestic sheep (*Ovis aries*) [29]. The ECM provides structures necessary for the connection between cells and directs cellular functions in the cardiovascular system. A continuous remodeling of ECM in cardiac valves is essential since the normal functional valve is subjected to mechanical and blood flow-induced shear stress. An altered ECM organization was observed in calcific aortic valve disease, characterized by severe calcification [30]. The ion homeostasis and related pathways are also upregulated in AVE cells. A transcriptome profiling of normal, stenotic, and regurgitant human valves also shows the enrichment of ion regulation in valvular endothelial cells [31]. The regulation of ions such as calcium might be an integral part of the healthy function of the extracellular matrix of valves [32].

Biological regulations related to proteins were upregulated in EE cells. The signaling pathways such as cytokine signaling in the immune system, signaling by PDGF, and interleukins were enriched in EE cells. A blood–heart barrier made by the endocardial endothelial cells can modulate the function of cardiomyocytes by sensory and endocrine roles. Endocardial endothelial can secrete signaling molecules such as angiopoietin, vascular endothelial growth factor (VEGF), endothelin, angiotensin-II, prostaglandins, nitric oxide, and fibroblast growth factor [33,34].

The endothelial cells maintain the hemostasis function by releasing different fibrinolytic proteins, anticoagulants, vasodilators, vasoconstrictors, and procoagulants [35]. In the present study, proteins related to hemostasis, platelet activation-signaling and aggregation, platelet degranulation, and response to elevated platelet cytosolic Ca^2+^ were enriched in both endothelial cells. Endothelial cells prevent thrombosis by different antiplatelet and anticoagulant mechanisms in normal physiological circumstances. Endothelial dysfunction can lead to atherogenesis as well as arterial thrombosis [36].

This study is the first proteome level comparison of endothelial cells isolated from the aortic valve and endocardium. Our results showed that most of the proteins and pathways are shared in both types of endothelial cells. In addition, we have observed several marker proteins in the AVE and EE cells. Our future objective will be to validate the cell surface markers using Western blots and fluorescently labeled antibody-based methods. We also believe that those markers will be helpful to develop a methodology to sort the AVE and EE cells from a heterogeneous group of cells using the fluorescence-activated cell sorting (FACS) method. The segregated group of specific endothelial cells will help us to understand pathogenic and normal physiological functions. 

We have detected several differentially expressed proteins in AVE and EE cells. The study’s primary limitation is that the physiological impact of those changed proteins was not explored further. Different types of functional studies such as shear-stress measurements and wound-healing assays are needed to link the protein levels difference to physiological functions of the endothelium in the future.

## 5. Conclusions

In conclusion, the proteome profile comparison of aortic valvular and endocardial endothelial cells suggests that these cells have a distinct phenotype. The biological functions, mechanical and shear stress, might determine the differences in the proteome of these two cells. The markers identified in both cells could be helpful for future studies targeted for a subpopulation of endothelial cells. The pathway analysis showed that metabolic process-related proteins and extracellular matrix-related proteins were enriched in valves. An enrichment of signaling pathways was observed in the endocardium. The hemostasis function-related proteins were increased in both endothelial cells. The present results provide a foundation for future studies to unravel normal physiology and different disease mechanisms that affect aortic valvular and endocardial endothelial cells.

## Data Availability

The proteomics data were uploaded in the ProteomeXchange Consortium via the PRIDE partner repository with the dataset identifier PXD025922.

## Supplementary Materials

The following are available online at https://www.mdpi.com/article/10.3390/genes12071005/s1, Figure S1: The rank plot of proteins in the endothelial cells detected by LC-MS analysis; Table S1: Table represents significantly changed proteins in aortic valvular and endocardial endothelium.
