# Supplementary material for: Proteomics Analysis Reveals Diverse Molecular Characteristics between Endocardial and Aortic-Valvular Endothelium
Source: Genes (Basel). 2021 Jun 30;12(7):1005. doi: 10.3390/genes12071005 (PMC8304717; doi:10.3390/genes12071005)
Supplement: Supplementary file 1 [file genes-12-01005-s001.zip › genes-1241940-supplementary.pdf]

# Supplementary Data

**Supplementary Table S1.** Table represents significantly changed proteins in Aortic valvular and Endocardial endothelium. The student's t-test was used for the differential expression analysis using Progenesis QI for Proteomics. A protein with a raw p-value < 0.05 and fold change > 1.5 was considered significant. AVE- Aortic valvular endothelium; EE- Endocardial endothelium.

| RefSeq Protein Accession | Gene Symbol | Description | Peptide count | Unique peptides | Anova (p) | q Value | Fold change | Up regulated Sample |
|---|---|---|---|---|---|---|---|---|
| NP_001026964 | SPARC | SPARC | 1 | 1 | 0.0005 | 0.0323 | 439.49 | AVE |
| XP_013849879 | CD44 | CD44 antigen | 6 | 3 | 0.0000 | 0.0003 | 197.24 | AVE |
| XP_020939373 | FARSA | Phenylalanine--tRNA ligase alpha subunit | 3 | 2 | 0.0112 | 0.1566 | 129.88 | AVE |
| XP_020925076 | COPG1 | Coatomer subunit gamma-1 | 4 | 3 | 0.0220 | 0.2217 | 43.10 | AVE |
| XP_001928917 | HKDC1 | Putative hexokinase HKDC1 | 8 | 4 | 0.0140 | 0.1782 | 42.03 | AVE |
| NP_001231275 | DNAJB11 | DnaJ homolog subfamily B member 11 | 1 | 1 | 0.0006 | 0.0323 | 37.77 | AVE |
| NP_001155875 | OTUB1 | Ubiquitin thioesterase OTUB1 | 2 | 1 | 0.0064 | 0.1252 | 11.69 | AVE |
| NP_008637 | COX2 | Cytochrome c oxidase subunit II | 1 | 1 | 0.0032 | 0.0855 | 6.27 | AVE |
| NP_999022 | TIMP1 | Metalloproteinase inhibitor 1 | 3 | 2 | 0.0029 | 0.0855 | 5.87 | AVE |
| XP_020943654 | ALDOA | Fructose-bisphosphate aldolase A | 21 | 1 | 0.0168 | 0.1961 | 5.55 | AVE |
| XP_020932236 | DDX4 | Probable ATP-dependent RNA helicase DDX4 | 3 | 2 | 0.0073 | 0.1291 | 5.45 | AVE |
| NP_001163993 | CCT7 | T-complex protein 1 subunit eta | 17 | 10 | 0.0191 | 0.2068 | 4.71 | AVE |

| | | | | | | | | |
|---|---|---|---|---|---|---|---|---|
| XP_005661703 | SEPTIN8 | Septin-8 X4 | 6 | 3 | 0.0097 | 0.1484 | 4.70 | AVE |
| XP_005666094 | XPO5 | Exportin-5 | 2 | 2 | 0.0187 | 0.2058 | 4.12 | AVE |
| XP_005661444 | DAZAP1 | DAZ-associated protein 1 | 4 | 3 | 0.0175 | 0.2020 | 3.78 | AVE |
| XP_020943629 | MED23 | Mediator of RNA polymerase II transcription subunit 23 | 2 | 1 | 0.0214 | 0.2192 | 3.33 | AVE |
| XP_001927130 | NT5E | 5'-nucleotidase | 3 | 3 | 0.0016 | 0.0592 | 3.28 | AVE |
| XP_005673382 | DBNL | Drebrin-like protein | 5 | 2 | 0.0000 | 0.0009 | 3.02 | AVE |
| XP_020922812 | COL1A1 | Collagen alpha-1(I) chain | 40 | 30 | 0.0059 | 0.1234 | 2.77 | AVE |
| NP_001072131 | PARK7 | Parkinson disease protein 7 | 6 | 4 | 0.0044 | 0.0990 | 2.73 | AVE |
| NP_001103415 | CDH13 | Cadherin-13 | 3 | 3 | 0.0048 | 0.1062 | 2.38 | AVE |
| NP_001230731 | RAB8A | Ras-related protein Rab-8A | 7 | 1 | 0.0160 | 0.1907 | 2.28 | AVE |
| XP_020923404 | C1QBP | Complement component 1 Q subcomponent-binding protein | 2 | 2 | 0.0106 | 0.1542 | 2.25 | AVE |
| XP_003121745 | SERPINB2 | Plasminogen activator inhibitor 2 | 3 | 2 | 0.0002 | 0.0213 | 2.13 | AVE |
| XP_020944376 | ATP1A1 | Sodium/potassium-transporting ATPase subunit alpha-1 | 34 | 11 | 0.0005 | 0.0323 | 1.94 | AVE |
| NP_001001542 | ATP1B1 | Sodium/potassium-transporting ATPase subunit beta-1 | 3 | 1 | 0.0077 | 0.1295 | 1.79 | AVE |
| NP_001230736 | FKBP10 | Peptidyl-prolyl cis-trans isomerase FKBP10 | 12 | 6 | 0.0112 | 0.1566 | 1.63 | AVE |
| NP_001127826 | ACTR2 | Actin-related protein 2 | 5 | 2 | 0.0179 | 0.2026 | 1.57 | AVE |
| XP_020952581 | YB | Nuclease-sensitive element-binding protein 1 | 7 | 5 | 0.0069 | 0.1286 | 1.50 | AVE |
| NP_001161122 | MAT2A | S-adenosylmethionine synthase type-2 | 1 | 1 | 0.0022 | 0.0729 | 2907.01 | EE |

| | | | | | | | | |
|---|---|---|---|---|---|---|---|---|
| NP_001033715 | AKR1C4 | Aldo-keto reductase family 1_ member C-like 1 | 12 | 3 | 0.0061 | 0.1236 | 5.96 | EE |
| XP_020932718 | NPR3 | Atrial natriuretic peptide receptor 3 | 1 | 1 | 0.0000 | 0.0003 | 5.60 | EE |
| NP_001156878 | PROCR | Endothelial protein C receptor | 9 | 9 | 0.0100 | 0.1484 | 3.45 | EE |
| NP_998981 | ICAM1 | Intercellular adhesion molecule 1 | 4 | 2 | 0.0043 | 0.0990 | 3.35 | EE |
| NP_999166 | TFRC | Transferrin receptor protein 1 | 9 | 4 | 0.0155 | 0.1883 | 2.62 | EE |
| XP_005663935 | CD63 | CD63 antigen | 2 | 1 | 0.0000 | 0.0024 | 2.54 | EE |
| NP_001231465 | THBS1 | Thrombospondin-1 | 32 | 21 | 0.0122 | 0.1605 | 2.34 | EE |
| XP_003131576 | KPNB1 | Importin subunit beta-1 | 18 | 12 | 0.0121 | 0.1605 | 2.26 | EE |
| XP_005670835 | NEFH | Neurofilament heavy polypeptide | 9 | 3 | 0.0037 | 0.0900 | 2.24 | EE |
| XP_003483583 | PGAM1 | Phosphoglycerate mutase 1 | 12 | 10 | 0.0031 | 0.0855 | 2.22 | EE |
| XP_020937128 | MCM5 | DNA replication licensing factor MCM5 | 4 | 4 | 0.0058 | 0.1232 | 2.08 | EE |
| XP_020938415 | CORO1B | Coronin-1B | 1 | 1 | 0.0211 | 0.2192 | 2.01 | EE |
| XP_013836290 | PECAM1 | Platelet endothelial cell adhesion molecule | 7 | 5 | 0.0242 | 0.2394 | 1.96 | EE |
| XP_005654650 | RAPGEF1 | Rap guanine nucleotide exchange factor 1 | 3 | 1 | 0.0120 | 0.1605 | 1.88 | EE |
| NP_001116685 | VAPB | Vesicle-associated membrane protein-associated protein B | 4 | 2 | 0.0000 | 0.0023 | 1.84 | EE |
| XP_003124995 | CAPG | Macrophage-capping protein | 11 | 8 | 0.0156 | 0.1883 | 1.81 | EE |
| XP_020947038 | LOC110260611 | Uncharacterized protein LOC110260611 | 1 | 1 | 0.0081 | 0.1303 | 1.80 | EE |

| | | | | | | | | |
|---|---|---|---|---|---|---|---|---|
| NP_001231864 | TALDO1 | Transaldolase | 7 | 3 | 0.0002 | 0.0221 | 1.71 | EE |
| XP_020929069 | LOC100155 138 | tubulin alpha-8 chain | 22 | 1 | 0.0025 | 0.0788 | 1.71 | EE |
| NP_001001636 | RPL32 | 60S ribosomal protein L32 | 4 | 4 | 0.0100 | 0.1484 | 1.61 | EE |
| NP_001132944 | PSMA6 | Proteasome subunit alpha type-6 | 6 | 3 | 0.0008 | 0.0405 | 1.61 | EE |
| XP_003483854 | BASP1 | Brain acid soluble protein 1 | 14 | 13 | 0.0002 | 0.0213 | 1.57 | EE |
| XP_003132638 | CCDC50 | Coiled-coil domain-containing protein 50 | 5 | 2 | 0.0005 | 0.0323 | 1.57 | EE |
| XP_020956671 | WDR1 | WD repeat-containing protein 1 | 19 | 9 | 0.0132 | 0.1712 | 1.55 | EE |

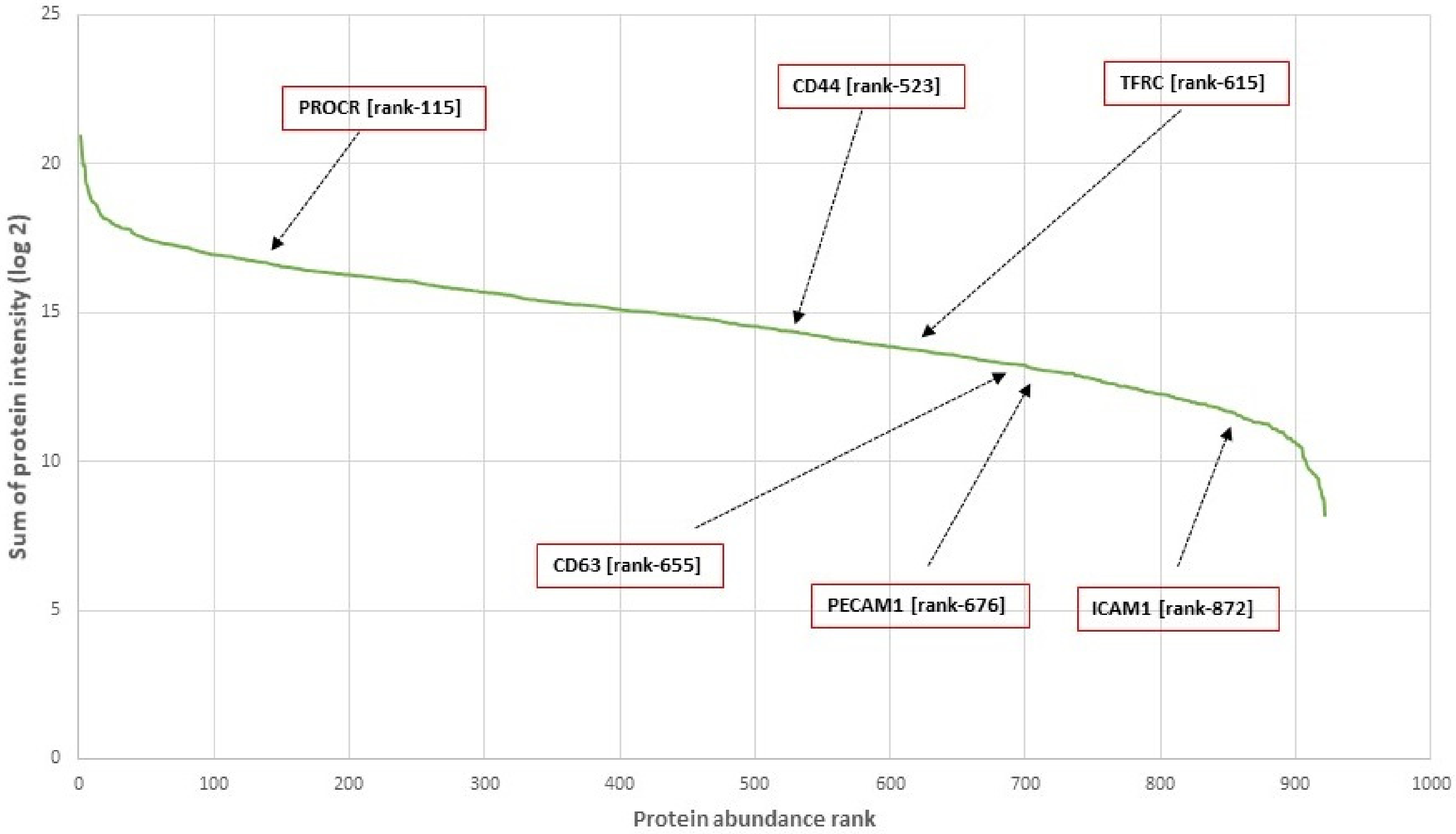


**Supplementary Figure S1.** The rank plot of proteins in the endothelial cells detected by LC-MS analysis using the sum of the protein intensity. Cell surface marker proteins were highlighted in the figure.
